# PRAME protein expression in DICER1‐related tumours

**DOI:** 10.1002/cjp2.293

**Published:** 2022-08-20

**Authors:** Paul S Thorner, Anne‐Sophie Chong, Javad Nadaf, Naciba Benlimame, Paula Marrano, Rose Chami, Lili Fu, William D Foulkes

**Affiliations:** ^1^ Department of Laboratory Medicine and Pathobiology University of Toronto Toronto Ontario Canada; ^2^ Department of Human Genetics McGill University Montreal, QC Canada; ^3^ Cancer Axis, Lady Davis Institute for Medical Research Jewish General Hospital Montreal, QC Canada; ^4^ Cancer Research Program Research Institute of the McGill University Health Centre Montreal, QC Canada; ^5^ Research Pathology Facility Lady Davis Institute, Jewish General Hospital Montreal, QC Canada; ^6^ Division of Pathology Hospital for Sick Children Toronto Ontario Canada; ^7^ Department of Pathology McGill University Health Centre, McGill University Montreal, QC Canada; ^8^ Gerald Bronfman Department of Oncology McGill University Montreal, QC Canada

The authors have identified some minor errors in the published version of the S1, S2 for this paper. Corrected tables are provided here and supersede the previously published versions. The authors apologise for any inconvenience caused.

**Table S1:** Patients with multiple lesions included in the study

Patient	Specimen type and number	Germline *DICER1* variant	Somatic *DICER1* variant
**Patient 1**	MNG 3	c.3907_3908delCT, p.Leu1303ValfsTer4	c.5125G>A, p.Asp1709Asn
	Cervical embryonal RMS 3	c.3907_3908delCT, p.Leu1303ValfsTer4	c.5113G>A, p.Glu1705Lys
**Patient 2**	MNG 6	c.4566_4570dupCTTTG, p.Val1524AlafsTer38	c.5438A>T, p.Glu1813Val
	CN 6	c.4566_4570dupCTTTG, p.Val1524AlafsTer38	None
	CN 7	c.4566_4570dupCTTTG, p.Val1524AlafsTer38	c.5113G>A, p.Glu1705Lys
	Neuroblastoma 1	None	None
**Patient 3**	MNG 8	c.4207‐41_5096+1034del, p.Thr1403_Glu1788del	c.5113G>C, p.Glu1705Gln
	SLCT of ovary, moderately differentiated 11	c.4207‐41_5096+1034del, p.Thr1403_Glu1788del	c.5437G>C, p.Glu1813Gln
**Patient 4**	Thyroid carcinoma (follicular variant of papillary) 2	c.5437G>C, p.Glu1813Gln (MOSAIC)	LOH
	PPB Type Ir 1	c.5437G>C, p.Glu1813Gln (MOSAIC)	None
	SLCT of ovary, moderately differentiated 7	c.5437G>C, p.Glu1813Gln (MOSAIC)	c.4626_4626delG, p.Gln1542HisfsTer18
	SLCT of ovary, poorly differentiated **5**	c.5437G>C, p.Glu1813Gln (MOSAIC)	LOH
**Patient 5**	CN 5	c.4754C>G, p.Ser1585Ter	c.5438A>G, p.Glu1813Gly
	CN 9	c.4754C>G, p.Ser1585Ter	c.5439G>T, p.Glu1813Asp
	Pineoblastoma 1	c.4754C>G, p.Ser1585Ter	None
**Patient 6**	Intracranial spindle cell sarcoma 1	Full gene deletion	c.5125G>A, p.Asp1709Asn
	CBME 1	Full gene deletion	c.5437G>A, p.Glu1813Lys
**Patient 7**	PPB Type I 3	c.5125G>A, p.Asp1709Asn (MOSAIC)	None
	PPB Type II 3	c.5125G>A p.Asp1709Asn (MOSAIC)	None
	NCMH 2	c.5125G>A, p.Asp1709Asn (MOSAIC)	None
**Patient 8**	CN 2	c.2379T>G, p.Tyr793Ter	c.5428G>T, p.Asp1810Tyr
	PPB Type Ir 2	c.2379T>G, p.Tyr793Ter	c.5439G>T , p.Glu1813Asp
**Patient 9**	PPB Type II 2	c.5394delA, p.Glu1799LysfsTer39	c.5113G>A, p.Glu1705Lys
	NCMH 1	c.5394delA, p.Glu1799LysfsTer39	c.5439G>T, p.Glu1813Asp
**Patient 10**	CN 3	c.5439G>C, p.Glu1813Asp (MOSAIC)	None
	PPB I 1	c.5439G>C, p.Glu1813Asp (MOSAIC)	None

# Refer to Table S2 for complete listing and numbering of specimens

PPB = pleuropulmonary blastoma

RMS = rhabdomyosarcoma

NCMH = nasal chondromesenchymal hamartoma

CN = cystic nephroma

MNG = multinodular goitre

SLCT = Sertoli‐Leydig cell tumour

CBME = ciliary body medulloepithelioma

**Table S2:** *DICER1* variants identified in the lesions included in the study

Diagnosis	Specimen number	Germline *DICER1* variant	Somatic *DICER1* variant
**Multinodular goitre**	1	c.2805‐1G>T, p.Tyr936_Arg996del	c.5113G>A, p.Glu1705Lys^
	2	c.2516C>T, p.Ser839Phe	c.5428G>T, p.Asp1810Tyr^
	3	c.3907_3908delCT, p.Leu1303ValfsTer4	c.5125G>A, p.Asp1709Asn
	4	c.3907_3908delCT, p.Leu1303ValfsTer4	c.5065C>T, p.His1689Tyr^
	5	c.3270‐6_4051‐1280delinsG, p.Tyr1091SerTer28	c.5126A>G, p.Asp1709Gly^
	6	c.4566_4570dupCTTTG, p.Val1524AlafsTer38	c.5438A>T, p.Glu1813Val^
	7	c.4407_4410delTTCT, p.Ser1470LeufsTer19	c.5126A>G, p.Asp1709Gly
	8	c.4207‐41_5096+1034del, p.Thr1403_Glu1788del	c.5113G>C, p.Glu1705Gln
	9	c.4207‐41_5096+1034del, p.Thr1403_Glu1788del	c.5429A>T, p.Asp1810Val
	10	c.4207‐41_5096+1034del, p.Thr1403_Glu1788del	c.5126A>G, p.Asp1709Gly
	11	c.2805‐1G>T, p.Tyr936_Arg996del	None
**Thyroid carcinoma (follicular variant of papillary)**	1	c.3579_3580delCA, p.Asn1193LysfsTer41	c.5438A>G, p.Glu1813Gly
	2	c.5437G>C, p.Glu1813Gln (MOSAIC)	LOH
**Ciliary body medulloepithelioma**	1	Full gene deletion	c.5437G>A, p.Glu1813Lys
**Intracranial spindle cell sarcoma**	1	Full gene deletion	c.5125G>A, p.Asp1709Asn
**Nasal chondromesenchymal hamartoma**	1	c.5394delA, p.Glu1799LysfsTer39	c.5439G>T, p.Glu1813Asp
	2	c.5125G>A, p.Asp1709Asn (MOSAIC)	None
**Pineoblastoma**	1	c.4754C>G, p.Ser1585Ter	None
	2	c.1498A>T, p.Lys500Ter	LOH
**Sertoli‐Leydig cell tumour of ovary, moderately differentiated**	1	c.5018_5021delTCAA, p.Ile1673ThrfsTer31	c.5125G>A, p.Asp1709Asn
	2	c.2457C>G, p.Ile813_Tyr819del	c.5437G>A, p.Glu1813Lys
	3	c.3611_3616delACTACAinsT, p.Tyr1204LeufsTer29	c.5113G>A, p.Glu1705Lys
	4	None	c.5439G>C, p.Glu1813Asp
	5	c.1532_1533delAT, p.His511ArgfsTer16	c.5438A>C, p.Glu1813Ala
	6	c.1525C>T, p.Arg509Ter	c.5125G>A, p.Asp1709Asn
	7	c.5437G>C, p.Glu1813Gln (MOSAIC)	c.4626_4626delG, p.Gln1542HisfsTer18
	8	None	c.5113G>A, p.Glu1705Lys
			c.734+3A>G
	9	c.3300_3301insA, p.Ala1101IfsTer3	c.5437G>A, p.Glu1813Lys
	10	None	c.5125G>A, p.Asp1709Asn
			c.3080_3081insCT, p.Gln1028CysfsTer39
	11	c.4207‐41_5096+1034del, p.Thr1403_Glu1788del	c.5437G>C, p.Glu1813Gln
	12	c.1532_1533delAT, p.His511ArgfsTer16	c.5429A>T, p.Asp1810Val
	13	c.1174C>T, p.Arg392Ter	c.5425G>A, p.Gly1809Arg
**Sertoli‐Leydig cell tumour of ovary, poorly differentiated**	1	c.3300_3301insA, p.Ser1101IlefsTer3	c.5113G>A, p.Glu1705Lys
	2	c.1532_1533delAT, p.His511ArgfsTer16	c.5113G>A, p.Glu1705Lys
	3	None	c.2808T>G, p.Tyr936Ter
			c.5125G>A, p.Asp1709Asn
	4	None	c.5437G>A, p.Glu1813Lys
			c.5487_5490delAGTC, p.Val1830GlyfsTer7
	5	c.5437G>C, p.Glu1813Gln (MOSAIC)	LOH
**Adult pulmonary blastoma**	1	None	c.5125G>A, p.Asp1709Asn
			c.1668_1668delC, p.Ile557SerfsTer5
	2	None	c.1232C>A, p.Ser411Ter
			c.5425G>A, p.Gly1809Arg
	3	None	c.5189_5198delGGGTCCTGAC, p.Gly1730GlufsTer18
			c.5438A>T, p.Glu1813Val
**PPB Type I**	1	c.5439G>C, p.Glu1813Asp (MOSAIC)	None
	2	c.3293G>A, p.Trp1098Ter	c.5437G>A, p.Glu1813Lys
	3	c.5125G>A, p.Asp1709Asn (MOSAIC)	None
	4	c.4407_4410TTCT, p.Ser1470LeufsTer19	c.5125G>A, p.Asp1709Asn
**PPB Type Ir**	1	c.5437G>C, p.Glu1813Gln (MOSAIC)	None
	2	c.2379T>G, p.Tyr793Ter	c.5439G>T, p.Glu1813Asp
	3	c.2062C>T, p.Arg688Ter	c.5125G>A, p.Asp1709Asn
**PPB Type II**	1	c.4407_4410delTTCT, p.Ser1470LeufsTer19	c.5425G>A, p.Gly1809Arg
	2	c.5394delA, p.Glu1799LysfsTer39	c.5113G>A, p.Glu1705Lys
	3	c.5125G>A, p.Asp1709Asn (MOSAIC)	None
**PPB Type III**	1	c.940_941delGG, p.Gly314ThrfsTer6	c.5113G>A, p.Glu1705Lys
	2	None	c.5125G>A, p.Asp1709Asn
	3	c.2040+1G>T	c.5425G>A, p.Gly1809Arg
	4	Not available^#^	c.1269_1270delAA, p.Glu423AspfsTer2
			c.5439G>T, p.Glu1813Asp
**Cystic nephroma**	1	c.2379T>G, p.Tyr793Ter	c.5425G>A, p.Gly1809Arg
	2	c.2379T>G, p.Tyr793Ter	c.5428G>T, p.Asp1810Tyr
	3	c.5439G>C, p.Glu1813Asp (MOSAIC)	None
	4	None	c.5125G>A, p.Asp1709Asn
**Cystic nephroma**	5	c.4754C>G, p.Ser1585Ter	c.5438A>G, p.Glu1813Gly
	6	c.4566_4570dupCTTTG, p.Val1524AlafsTer38	None
	7	c.4566_4570dupCTTTG, p.Val1524AlafsTer38	c.5113G>A, p.Glu1705Lys
	8	c.4566_4570dupCTTTG, p.Val1524AlafsTer38	None
	9	c.4754C>G, p.Ser1585Ter	c.5439G>T, p.Glu1813Asp
**Wilms tumour**	1	c.912_919dup, p.Arg307GlnfsTer8	c.4031C>T, p.Ser1344Leu
	2	c.1306dupT, p.Ser436PhefsTer41	c.5138A>C, p.Asp1713Ala
**Cystic hepatic neoplasm**	1	c.4007delC, p.Pro1336LeufsTer11	c.5113G>C, p.Glu1705Gln
**Paratesticular tumour of probable Müllerian origin**	1	c.4308_4311del, p.Ala1436fs	c.5114A>T, p.Glu1705Val
**Embryonal RMS of ovary**	1	c.1196_1197dupAG, p.Trp400SerfsTer59	c.5425G>A, p.Gly1809Arg
**Embryonal RMS of cervix**	1	c.2457C>G, p.Ile813_Tyr819del	c.5439G>T, p.Glu1813Asp
	2	c.3611_3616delACTACAinsT, p.Tyr1204LeufsTer29	c.5438A>G, p.Glu1813Gly
	3	c.3907_3908delCT, p.Leu1303ValfsTer4	c.5113G>A, p.Glu1705Lys
	4	c.3535_3538delTCTT, p.Ser1179ThrfsTer12	c.5437G>A, p.Glu1813Lys
	5	None	c.2062C>T, p.Arg688Ter
			c.5438A>G, p.Glu1813Gly
**Anaplastic sarcoma of kidney** *	1	None	c.5439G>T, p.Glu1813Asp
			LOH
	2	c.2062C>T, p.Arg688Ter	c.5425G>A, p.Gly1809Arg

LOH – Loss of heterozygosity

* These two cases were not included in the TMA but full‐face sections were stained separately and scored using the same system as for the TMA

^#^ Two variants were identified in testing of PPB‐III 4. No germline DNA was available to determine whether the truncating variant was germline

^^^ These MNGs all had more than one somatic hit, here only one of the possible hotspot variants is included. We cannot be sure which one was present in the tissue punch that was used for the TMA.

